# Adenine Nucleotide Translocase 1 Expression Modulates the Immune Response in Ischemic Hearts

**DOI:** 10.3390/cells10082130

**Published:** 2021-08-19

**Authors:** Fatih Yergöz, Julian Friebel, Nicolle Kränkel, Ursula Rauch-Kroehnert, Heinz-Peter Schultheiss, Ulf Landmesser, Andrea Dörner

**Affiliations:** 1 Department of Cardiology, Charité—Universitätsmedizin Berlin, Corporate Member of Freie Universität Berlin, Humboldt-Universität zu Berlin and Berlin Institute of Health, 12200 Berlin, Germany; Fatih.Yergoez@charite.de (F.Y.); Julian.Friebel@charite.de (J.F.); Nicolle.Kraenkel@charite.de (N.K.); Ursula.Rauch@charite.de (U.R.-K.); Ulf.Landmesser@charite.de (U.L.); 2 Institute of Health Center for Regenerative Therapies (BCRT), Charité—Universitätsmedizin Berlin, 13353 Berlin, Germany; 3 DZHK (German Centre for Cardiovascular Research), Partner Site Berlin, 10785 Berlin, Germany; 4 Institute for Cardiac Diagnostics and Therapy (IKDT), 12203 Berlin, Germany; Heinz-Peter.Schultheiss@ikdt.de

**Keywords:** adenine nucleotide translocase, mitochondria, immune response, cytokine, macrophages, myocardial infarction, ischemic cardiomyopathy

## Abstract

Adenine nucleotide translocase 1 (ANT1) transfers ATP and ADP over the mitochondrial inner membrane and thus supplies the cell with energy. This study analyzed the role of ANT1 in the immune response of ischemic heart tissue. Ischemic ANT1 overexpressing hearts experienced a shift toward an anti-inflammatory immune response. The shift was characterized by low interleukin (IL)-1β expression and M1 macrophage infiltration, whereas M2 macrophage infiltration and levels of IL-10, IL-4, and transforming growth factor (TGFβ) were increased. The modulated immune response correlated with high mitochondrial integrity, reduced oxidative stress, low left ventricular end-diastolic heart pressure, and a high survival rate. Isolated ANT1-transgenic (ANT1-TG) cardiomyocytes expressed low levels of pro-inflammatory cytokines such as IL-1α, tumor necrosis factor α, and TGFβ. However, they showed increased expression and cellular release of anti-inflammatory immunomodulators such as vascular endothelial growth factor. The secretome from ANT1-TG cardiomyocytes initiated stress resistance when applied to ischemic wild-type cardiomyocytes and endothelial cells. It additionally prevented macrophages from expressing pro-inflammatory cytokines. Additionally, ANT1 expression correlated with genes that are related to cytokine and growth factor pathways in hearts of patients with ischemic cardiomyopathy. In conclusion, ANT1-TG cardiomyocytes secrete soluble factors that influence ischemic cardiac cells and initiate an anti-inflammatory immune response in ischemic hearts.

## 1. Introduction

Ischemic heart disease is a leading cause of death worldwide [1]. Therefore, a better understanding of the regulatory processes in infarcted hearts is crucial in response to ischemic stress. Myocardial intercellular crosstalk coordinates the tissue response in concert with the immune system. The inflammatory response to acute myocardial infarction (MI) is critical for determining the infarct size and left ventricular remodeling. Furthermore, inflammation is a significant process involved in disease progression. In the infarct zone, dying cardiac cells release signaling molecules, such as cytokines, chemokines, heat shock proteins, and reactive oxygen species, which trigger the immune response. Macrophages are the first most numerous immune cells that infiltrate the infarcted myocardium [2]. Macrophages display functionally heterogeneous phenotypes, spanning between the pro-inflammatory M1-type and the anti-inflammatory and pro-resolving M2-type. The initial pro-inflammatory phase that follows infarction serves to promote the clearance of debris and dying cells and is driven by M1-type macrophages. M2-type macrophages increasingly contribute to the regenerative stage, releasing vascular endothelial growth factor (VEGF) to promote vascularization and transforming growth factor (TGFβ) to promote scar formation. The release of anti-inflammatory cytokines by M2-like macrophages, including interleukin (IL)-10, further shapes the local immune profile.

Cardiac energy metabolism, especially mitochondrial processes, is currently discussed to significantly influence the immune response after tissue damage [3]. Mitochondria are the primary producers of energy; consequently, they affect all energy-consuming processes in the cell. Processes such as metabolic pathways, amino acid metabolism, antioxidant systems, mtDNA, mitophagy, and mtROS, all driven by mitochondria, are crucial for immunological reactions [4]. Thus, coordinated interactions between mitochondria and both intra- and extracellular processes require active communication to trigger a suitable inflammatory response. 

Adenine nucleotide translocase (ANT) is the most highly expressed mitochondrial protein, comprising 10% of the total protein content in cardiac mitochondria [5]. ANT facilitates the transfer of intramitochondrial ATP and extramitochondrial ADP across the inner mitochondrial membrane, linking energy-producing and energy-consuming processes [6]. In addition, ANT modulates the opening of the mitochondrial permeability transition pore, which ultimately mediates oxidative stress, and apoptotic and necrotic processes [7]. Moreover, ANT is also localized in the cell membrane, influencing cell signaling by interacting with the cell adhesion molecule L1 [8,9]. 

ANT1 is the predominant ANT isoform expressed in the heart [10]. Therefore, ANT1 mutations and reductions in myocardial ANT1 expression are associated with complex human diseases, accompanied by severe cardiac impairment [11,12]. Conversely, our previous work demonstrated that heart-specific ANT1 overexpression resulted in preserved ANT function in stressed cardiomyocytes, which had distinctive cardioprotective effects in various experimental models of heart disease [13,14,15]. The increased secretion of the heat shock protein, HSP27, belongs to these cardioprotective effects in ischemic ANT1-TG hearts [16]. Extracellular HSP27 signals via TLR4 and modulates anti-inflammatory processes [16,17]. Based on ANT’s central role in mitochondrial function and its regulating effect on intercellular communication, we investigated whether ANT1 overexpression modulates the myocardial immune response after heart infarction.

## 2. Materials and Methods

### 2.1. Animals

Heart-specific ANT1-TG Sprague Dawley rats were previously described in detail by Walther et al. [13]. MI was induced in male WT and heart-specific transgenic ANT1 overexpressing rats (WT_MI_ and ANT1-TG_MI_, 4–6 months old) for 24 h by permanent ligation of the left descending coronary artery (LAD) during a previous study [15]. In control groups (WT_sham_ and ANT1-TG_sham_), a suture was brought under the LAD without performing a ligature. Hemodynamic data and infarct size have been determined in the earlier study. All studies were approved by the international guidelines of Directive 2010/63/EU established by the European Parliament and the institutional animal care committee (G0158/08; T0449/08). All studies are in accordance with the ethical standards established by the 1964 Declaration of Helsinki and all later amendments.

### 2.2. Protein Isolation

Frozen infarcted heart tissue or cultured cardiomyocytes were lysed in ice-cold lysis buffer (Cell Signaling, Boston, MA, USA), containing PhosSTOP phosphatase inhibitors and a complete mini protease inhibitor (both from Roche Diagnostics Deutschland GmbH, Mannheim, Germany), according to the manufacturer’s recommendations. Lysates were centrifuged at 16,000× *g* for 30 min at 4 °C. Protein concentrations were determined by bicinchoninic acid (BCA) assay (Pierce, Bonn, Germany).

### 2.3. Western Blot Analysis

Mitochondria and cytosolic protein were isolated from the heart, according to the method of Smith [18]. The protein concentration was determined using the bicinchoninic acid test (Pierce, Bonn, Germany). Equal amounts of protein samples were separated on 4–12% polyacrylamide mini gels (NuPAGE^®^ Novex Bis-Tris-Gels, Life Technologies, Carlsbad, USA) and transferred onto polyvinylidene fluoride membranes (BioRAD, Munich, Germany). Five to six samples from two groups (ANT1-TG_sham_ vs. WT_sham_, ANT1-TG_MI_ vs. ANT1-TG_MI_, or WT_MI_, vs. WT_sham_) were applied on one mini gel. The membranes were blocked for 1 h with 5% dry non-fat milk in Tris-buffered saline with 0.1% Tween 20 (TBST) at room temperature (RT). Membranes were incubated overnight at 4 °C with primary antibodies against IL-1β (Cat.No.: BYT-ORB101745, Biozol Diagnostica Vertrieb GmbH, Eching, Germany) tumor necrosis factor α (TNFα) (Cat.No.: GWB-BIGCA2, Genway, Biotech INC, San Diego, USA), IL-6 (Cat.No.: AF506), IL-10 (Cat.No.: AF519), and IL-4 (Cat.No.: MAB504, R&D Systems, Wiesbaden-Nordenstadt, Germany), TGFβ1,2,3 (Cat.No.: sc-7892), cytochrome c (Cat.No.: sc-7159), β-actin (Cat.No.: sc-47778), and HSP27 (Cat.No.: sc-1048, Santa Cruz Biotechnology, Heidelberg, Germany), VEGF-A (Cat.No.: MAB0294, Abnova, Taipeh, Taiwan), CD86 (Cat.No.: 91882, Abcam Berlin, Germany, [19]) and ANT1 [15] in blocking solution, followed by a three-time wash in TBST at RT. Incubation with the appropriate horseradish peroxidase-conjugated secondary antibody (Dako, Glostrup, Denmark) was performed in blocking solution for 2 h at RT. The membranes were washed three-times for 10 min each in TBST at RT. According to the manufacturer’s instructions, antibody binding was detected using a WesternBright Chemilumineszenz Substrate Sirius (Biozym Scientific GmbH, Hessisch Oldendorf, Germany). Signals were quantified using Multi Gauge V3.0 and normalized against β-actin. Using corresponding samples, data from the different blots were equalized and presented as percentages of WT controls.

### 2.4. Measuring Serum Peroxides

24 h after surgery, blood was collected and stored on ice. Serum was obtained by centrifugation at 1000× *g* for 10 min and stored at −80 °C. Serum peroxide levels were determined using the PeroXOquant^TM^ Quantitative Peroxide Assay Kit from ThermoScientific (Erembodegem, Belgium) as described by the manufacturer [15]. 

### 2.5. Immunohistochemistry

Total macrophage quantification in the infarct area was performed by immunohistochemistry using CD68 antibodies (Cat.No.: PA1518, Booster Bio, Pleasanton, CA, USA) as described in Klumpe et al. [15]. M2 macrophage staining was conducted with a CD206-specific antibody (Cat.No.: ab64693, Abcam, Cambridge, U.K.). Image quantification was performed using ImageJ software (NIH). CD68-positive cells and CD206-positive cells were microscopically quantified in a blinded manner as cells per high power field (HPF; 0.237 mm^2^). The numbers from 10–20 HPF were averaged. M1-macrophages were calculated as CD68-positive cells that were CD206-negative. Data are shown as percentages of sham-operated WT controls. Calculation of M1 macrophage levels were confirmed by Western blots using CD86 antibodies (see Section 2.3 and Figure S1d). 

### 2.6. Cardiomyocyte Isolation and Cytokine Treatment

Neonatal cardiomyocytes from age-matched, two- to three-day-old WT and ANT1-TG rats were isolated and cultured, as previously described [20]. Cardiomyocytes were starved for 12 h in a serum-free culture medium supplemented with 1% penicillin/streptomycin and 2 µM 5-fluoro-2′-deoxyuridine. Cardiomyocytes were treated with phosphate-buffered saline (PBS) as control, IL-1β (30 ng/mL), TGF-β1 (10 ng/mL), IL-4 (20 ng/mL), interferon (IFN)γ (30 ng/mL), TNFα (40 ng/mL), IFN-β (30 ng/mL), TNFβ (100 ng/mL), IL-6 (20 ng/mL), or IL-1α (10 ng/mL) for 24 h at 37 °C. Cells were washed three times with ice-cold PBS and cell pellet was immediately frozen at −80 °C.

### 2.7. Real-Time PCR

According to the manufacturer’s protocol, total RNA was extracted from cardiomyocytes using the TRIzol method (Thermo Fisher Scientific, Berlin, Germany). A total of 1 µg RNA was reverse transcribed into cDNA using M-MLV Reverse Transcriptase (Thermo Fisher Scientific, Berlin, Germany) in a total volume of 10 µL, according to the manufacturer’s recommendations. The gene expression of IL-1β, IL-1α, IL-6, IL-4, IL-10, TGF-β, TNF-α, VEGF, and ANT1 was determined using TaqMan^®^ assays (Thermo Fisher Scientific, Berlin, Germany) according to the manufacturer’s recommendations, and were normalized to hypoxanthine-guanine phosphoribosyltransferase (HPRT) or glyceraldehyde 3-phosphate dehydrogenase (GAPDH) expression levels. Relative gene expression was determined via the comparative C(t) (ΔΔCt) method.

### 2.8. Secretome Isolation

Neonatal cardiomyocytes were cultured in a starvation medium (serum-free Dulbecco’s modified Eagle medium (DMEM) supplemented with 1% penicillin/streptomycin and 2 µM 5-fluoro-2′-deoxyuridine), to obtain WT and ANT1-TG conditioned medium. Cells were cultured at 37 °C in a normoxic atmosphere (74% N_2_; 5% CO_2_; and 21% O_2_) for 24 h. After collection, the culture medium was centrifuged at 300× *g* for 10 min to remove viable floating cells and then at 4000× *g* for 20 min at 4 °C to remove cell debris. The medium was further clarified by low-protein-binding vacuum filtration using a 0.2 µm filter and concentrated by approximately 30-fold in centrifugal filter units (Amicon^®^ Ultra-15 filter), following the manufacturer’s instructions. Protein quantification was performed using a standard BCA assay (Thermo Fisher Scientific). According to the manufacturer’s instructions, the obtained secretome was freshly used for viability assays (see below). The VEGF levels in the secretome were determined using Proteome Profiler Rat Cytokine Array Kit (R&D Systems, Wiesbaden-Nordenstadt, Germany).

### 2.9. Endothelial Cells

Human aortic endothelial cells (HAEC) were purchased from Cell Systems (Troisdorf, Germany) and propagated in fully supplemented endothelial cell growth medium-2 (EGM-2) with 10% fetal calf serum (FCS, Lonza Cologne, Germany). HAECs were used for assays at passages 6–8. Ischemia was introduced by exchanging the buffer for an ischemia-mimetic solution (in mM: 125 NaCl, 8 KCl, 1.2 KH_2_PO_4_, 1.25 MgSO_4_, 1.2 CaCl_2_, 6.25 NaHCO_3_, 5 sodium lactate, and 20 HEPES, pH 6.6) [21].

### 2.10. Viability Assay

Cardiomyocytes and endothelial cells were seeded in white, opaque-walled, 96-well plates in their respective complete media. After 24 h, all media were removed, and the seeded cells were washed three times with PBS and cultured in serum-free medium with hypoxia (described in [20] or ischemic mimetic solution for an additional 24 h), respectively. Cells were treated with respective 5 µL serum-free cardiomyocyte or EGM-2 medium (control) or with 1 µg/mL or 10 µg/mL of WT- and ANT1-TG cardiomyocyte-derived secretome. Cell viability was measured using the CellTiter-Glo^®^ luminescent assay (Promega GmbH, Walldorf, Germany), following the manufacturer’s instructions, using a luminescent plate reader (Tecan, Männedorf, Switzerland).

### 2.11. THP1 Cells

THP1 (human acute monocytic leukemia cell) cells were differentiated into macrophages in 12-well dishes containing 0.5 mL of RPMI medium (10 mM Hepes, 1 mM Pyruvate, 1× Pen/Strep, and 50 pM β-mercaptoethanol) with 50 ng/mL phorbol 12-myristate 13-acetate (PMA) over 48 h. The supernatants were discarded, and the plates were washed three times with PBS. THP1 cells were treated with RPMI medium containing 5 µL fresh serum-free cardiomyocyte medium and 1 µg/mL WT- or ANT1-derived secretome, for 1 h at 37 °C. Cells were harvested, and cytokine expression was determined by real-time PCR.

### 2.12. ANT1 Co-Expression in Human Heart Tissue

Data from microarray assays were used, which were previously published in the NCBI Gene Expression Omnibus (GEO) database under the accession number GDS651, entitled “Heart failure arising from different etiologies” (https://www.ncbi.nlm.nih.gov, accessed on 10 August 2021). Data were generated from donors’ left ventricular heart tissue (*n* = 11), whose hearts could not be used for transplants, and patients with ischemic heart disease (ICM, *n* = 11), who underwent heart transplantation. Microarrays were normalized with the MAS5 method. ANT1 correlated genes were determined by Spearman correlation with a correlation factor r ≥ 0.6 and an adjusted *p*-value < 0.05 as cutoffs. Enrichment analyses of correlated genes were performed using bioinformatic programs as STRING [22] and REACTOME [23]. 

### 2.13. Data Presentation and Statistics

Data are presented relative to the values from corresponding control groups. The Mann–Whitney U test was used to evaluate differences between two groups. Kruskal-Wallis test was used for more than two preselected pairs of datasets followed by the Dunn’s test. Values are presented as the mean ± standard error of the mean. Correlation analyses were performed using Pearson’s correlation test. Differences were considered significant at values of *p* < 0.05. Statistical analyses were performed using GraphPad Prism version 6.0 (GraphPad Software, Inc., San Diego, CA, USA).

## 3. Results

### 3.1. Infarcted ANT1-TG Hearts Show a Modulated Cytokine Pattern 

Previous studies have demonstrated that infarcted hearts reduced ANT1 expression [15]; transgenic ANT overexpression compensated for decreased ANT1 protein and attenuated cardiac damage. Here, the immune response was analyzed in heart tissue from WT and ANT1-TG rats after MI to clarify whether ANT1 might play a direct causal role in modulating the myocardial immune profile.

**Figure 1:**
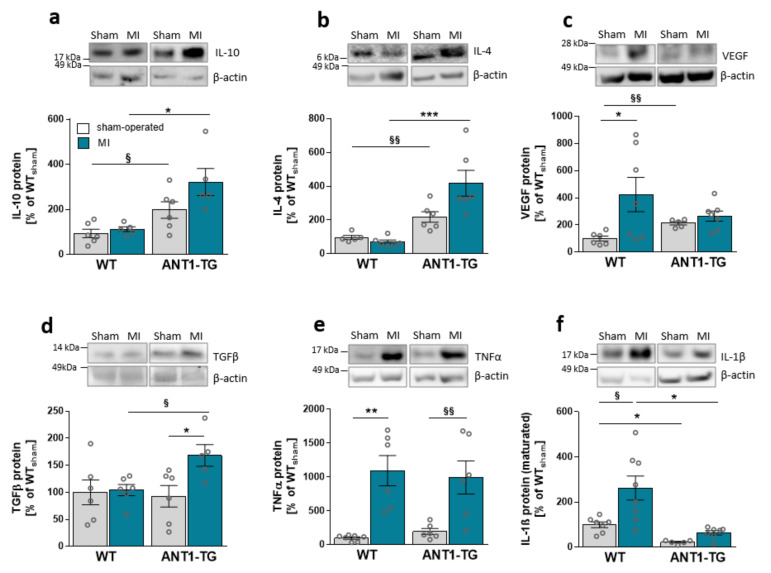
Cytokine expression pattern in sham-operated and infarcted myocardium derived from WT and ANT1-TG rats. Cytokine protein abundance of (**a**) IL-10, (**b**) IL-4, (**c**) VEGF-A, (**d**) TGFβ, (**e**) TNFα, and (**f**) IL-1β in sham-operated and infarcted heart tissue of WT and ANT1-TG rats. The upper panel shows representative Western blots, with β-actin as the internal control. * *p* < 0.05; ** *p* ≤ 0.01, *** *p* ≤ 0.001 calculated with the Kruskal–Wallis test, using the Dunn’s test as post hoc test; ^§^ *p* < 0.05; ^§§^ *p* < 0.01 calculated with Mann–Whitney U test. WT: wild-type rat; ANT1-TG: adenosine nucleotide translocase 1 transgenic rat; MI: myocardial infarction; IL: interleukin; VEGF: vascular endothelial growth factor; TGFβ: transforming growth factor β; and TNFα: tumor necrosis factor α.

Compared with sham-operated WT hearts, the protein levels of anti-inflammatory cytokines, such as IL-10 and IL-4, and growth factors, including VEGF, were elevated in sham-operated ANT1-TG hearts (Figure 1a–c). However, TGFβ and the pro-inflammatory TNFα was unchanged, and IL-1β was reduced (Figure 1e,d). 

After MI, ANT1-TG hearts produced more anti-inflammatory IL-10, IL-4, and pleiotropic TGFβ (Figure 1a,b,d). The protein level of TNFα was elevated to the same extent in both animal strains (Figure 1e). In contrast, pro-inflammatory IL-1β levels significantly increased in ischemic WT hearts but were only slightly elevated in ANT1-TG hearts (Figure 1f). Overall, ANT1-TG heart tissue showed a more anti-inflammatory cytokine expression pattern than WT hearts before and after infarction.

**Figure 2:**
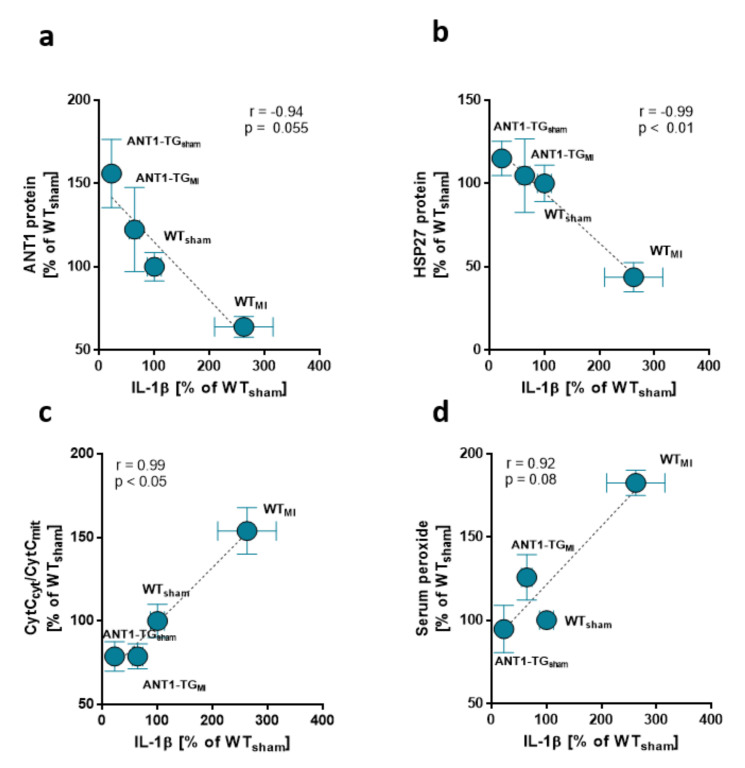
Low IL-1β expression correlates with mitochondrial integrity and low oxidative stress in ANT1-TG hearts. Mean IL-1β levels found in the sham and infarcted hearts of WT and ANT1-TG rats negatively correlated with the mean expression of (**a**) HSP27 and (**b**) ANT1 and were associated with (**c**) an increase in the ratio of cytosolic to mitochondrial cytochrome C (CytCcyt/CytCmit), a marker for mitochondrial function, and (**d**) serum peroxide levels [15]. Correlation coefficients and *p*-values determined by the Pearson’s correlation test are shown in the graphs, *n* = 5–6 for each group. IL: interleukin; WT: wild-type; ANT1-TG: adenosine nucleotide translocase 1 transgenic rat; HSP: heat shock protein.

ANT1-TG cardiomyocytes express and release increased amounts of HSP27, which exhibit anti-inflammatory and cell-protective properties [16,17]. Here, we demonstrated that high ANT1 and HSP27 expression levels negatively correlated with pro-inflammatory IL-1β protein levels (Figure 2a,b) in rat hearts. Reduced IL-1β protein expression was accompanied by a low ratio of cytosolic to mitochondrial cytochrome c (CytC_cyt_/CytC_mit_), an indicator of the inner mitochondrial membrane integrity (Figure 2c). Furthermore, low IL-1β levels correlated with reduced oxidative stress, as demonstrated by a low serum peroxide level (Figure 2d). Thus, the reduced expression of pro-inflammatory IL-1β was associated with retained mitochondrial function in ANT1-TG hearts compared with WT hearts.

### 3.2. Macrophage Pattern Shifts toward M2-Specific Macrophages

**Figure 3:**
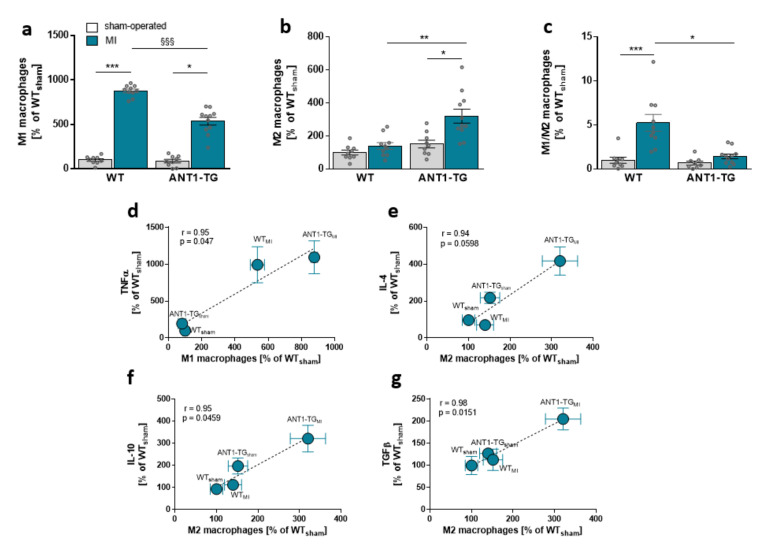
Myocardial macrophage levels and their relationships with immune modulator expression. (**a**) The percentages of M1-, (**b**) M2-specific macrophages, and (**c**) the M1/M2 macrophage ratio in sham-operated and infarcted (MI) hearts of WT and ANT1-TG rats. * *p* < 0.05, ** *p* ≤ 0.01, *** *p* ≤ 0.001 calculated with the Kruskal–Wallis test, using the Dunn’s test as post hoc test. §§§ *p* < 0.001 calculated with Mann–Whitney U test. The mean ± SEM of M1 macrophage levels found in the sham and infarcted hearts of WT and ANT1-TG rats positively correlated with the means of (**d**) TNFα. M2 macrophage levels were linked to (**e**) IL-4, (**f**) IL-10, and (**g**) TGFβ. Correlation coefficients (**r**) and *p*-values are shown in the graphs. WT: wild-type; ANT1-TG: adenosine nucleotide translocase 1 transgenic rats; SEM: standard error of the mean; TNFα: tumor necrosis factor α; IL: interleukin; TGFβ: transforming growth factor β.

ANT1 overexpression induces an anti-inflammatory cytokine pattern in transgenic hearts, introducing the possibility that the pattern of immunocompetent cells might differ between WT and ANT1-TG hearts. The level of total macrophages did not differ between ischemic WT and ANT1-TG hearts (Figure S1a,b). However, the proportion of pro-inflammatory M1 macrophages was lower in ischemic ANT1-TG than that in WT hearts (Figure 3a and Figure S1d). In contrast, the level of anti-inflammatory M2 macrophages increased in ANT1-TG hearts compared with WT hearts and resulted in a significant shift in the macrophage subtype pattern between WT and ANT1-TG hearts (Figure 3b,c and Figure S1c). 

Higher M1 macrophage levels were associated with TNFα expression (Figure 3d), whereas M2 macrophage levels correlated with IL-4, IL-10, and TGFβ protein levels in the myocardium of WT and ANT1-TG hearts (Figure 3e,g). 

**Figure 4:**
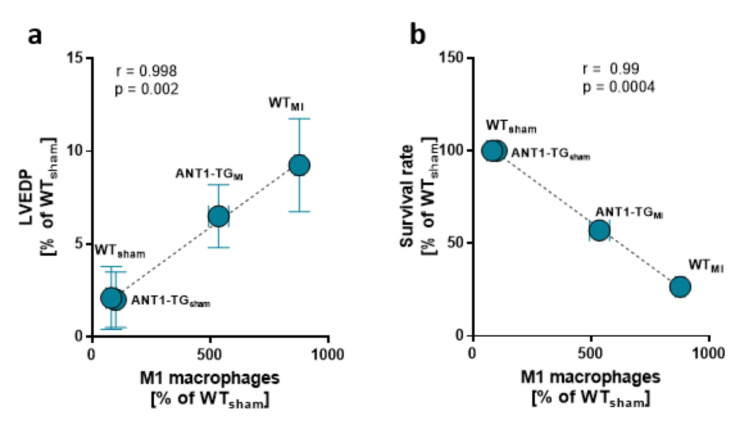
Myocardial macrophage levels and hemodynamics. The mean ± SEM of M1 macrophage levels found in the sham and infarcted hearts of WT and ANT1-TG rats was positively correlated with the means of (**a**) left ventricular end-diastolic pressure (LVEDP) and was negatively correlated with the (**b**) survival rate.

Diminished myocardial M1 macrophage infiltration correlated with reduced left ventricular end-diastolic pressure measured in the hearts and higher survival rates, linking the modulated immune response to myocardial function (Figure 4a,b).

### 3.3. Cytokine and ANT1 Transcription Influence Each Other in Cardiomyocytes

**Figure 5:**
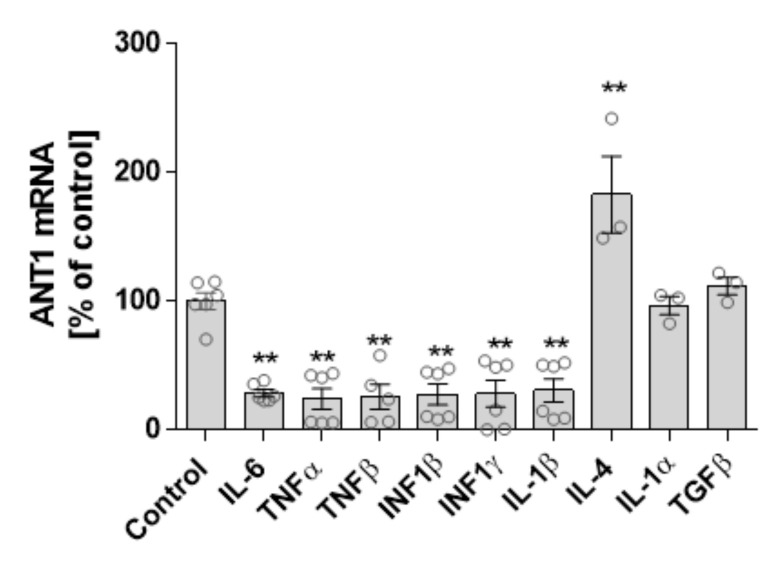
Relationship between ANT1 and cytokine transcription. Isolated neonatal rat cardiomyocytes were treated with various cytokines for 24 h. mRNA levels of treated cells were determined by quantitative PCR. Data are shown as mean ± SEM, ** *p* ≤ 0.01 vs. untreated control.

We analyzed the extent to which cytokine and ANT1 transcription depend on one another. Isolated rat cardiomyocytes were treated with various cytokines and interferons (INFs), and ANT1 mRNA expression was determined by quantitative PCR. Treatment with the pro-inflammatory cytokines IL-6, TNFα/β, INF1β/γ, and IL-1β significantly decreased ANT1 mRNA levels (Figure 5). In contrast, anti-inflammatory IL-4 boosted ANT1 transcription, whereas IL-1α and TGFβ did not affect ANT1 mRNA expression.

**Figure 6:**
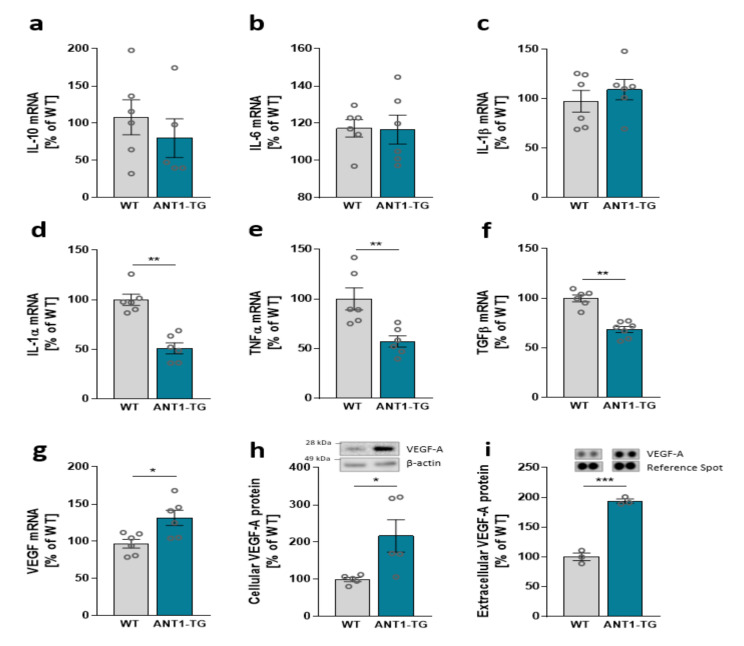
Cytokine mRNA expression levels of (**a**) IL-10, (**b**) IL-6, (**c**) IL-1β, (**d**) IL-1α, (**e**) TNFα, (**f**) TGFβ, and (**g**) VEGF in WT and ANT1-TG cardiomyocytes. (**h**) VEGF-A protein abundance in WT and ANT1-TG cardiomyocytes and (**i**) levels of VEGF protein released from the cardiomyocytes; upper panels show representative Western or dot blots. Data are presented as the mean ± SEM, * *p* ≤ 0.05, ** *p* ≤ 0.01, *** *p* ≤ 0.001 vs. WT cardiomyocytes.

Inversely, ANT1 overexpression modulates the cytokine expression pattern in isolated transgenic cardiomyocytes. Isolated WT and ANT1-TG cardiomyocytes expressed equal mRNA levels of IL-10, IL-6, and IL-1β (Figure 6a–c). However, the ANT1 overexpression resulted in reduced mRNA levels of IL-1α, TNFα, and TGFβ (Figure 6d–f), whereas VEGF mRNA increased in ANT1-TG cardiomyocytes (Figure 6g). Additionally, the increased transcription of VEGF resulted in elevated intracellular VEGF-A protein expression and secretion from ANT1-TG cardiomyocytes (Figure 6h,i). 

### 3.4. The Secretome of ANT1-TG Cardiomyocytes Affects Other Cardiac Cells

**Figure 7:**
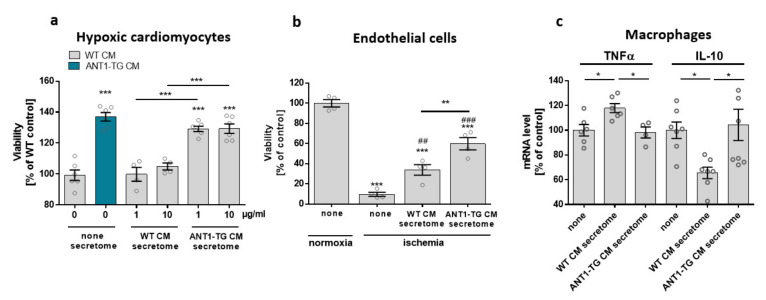
Treatment of cardiomyocytes, endothelial cells, and macrophage-like differentiated THP1 cells with the secretome derived from WT or ANT1-TG cardiomyocytes. (**a**) Hypoxic WT or ANT1-TG cardiomyocytes (CM) were cultured in the absence (none) or presence of secretomes (1 and 10 µg/mL) isolated from WT or ANT1-TG cardiomyocytes. Viability was assessed after 24 h (**b**) co-incubation of ischemic endothelial cells occurred with or without WT or ANT1-TG cardiomyocyte-derived secretome (1 µg/mL). Endothelial cell viability was assessed after 24 h. (**c**) Macrophage-like THP1 cells were treated with secretome (1 µg/µL) derived from WT and ANT1-TG cardiomyocytes for 1 h. TNFα and IL-10 mRNA levels were determined by qPCR. Data are shown as the mean ± SEM; * *p* ≤ 0.05, ** *p* ≤ 0.01, and *** *p* ≤ 0.001 vs. untreated controls or as shown, ## *p* < 0.01; ### *p* < 0.001 vs. ischemic untreated endothelial cells.

WT and ANT1-TG cardiomyocytes differed in the expression and release of signal modulating proteins such as VEGF and HSP27 [16], resulting in different secretome compositions. ANT1-TG cardiomyocytes were more resistant to hypoxic stress than WT cardiomyocytes (Figure 7a). Therefore, we tested whether this cell-protective information can be transferred from ANT1-TG cardiomyocytes to other cells using WT and ANT1-TG cardiomyocyte-derived secretomes. Hypoxic WT cardiomyocytes treated with the secretome obtained from WT cardiomyocytes (1 µg/mL and 10 µg/mL) did not affect cell viability, whereas the treatment with ANT1-TG cardiomyocyte secretome significantly increased cell survival. Furthermore, the ANT1-TG-derived secretome also elevated the cell viability of endothelial cells two times more than the secretome taken from WT cardiomyocytes (Figure 7b).

In addition, stimulation of macrophage-like THP1 cells with the secretome derived from WT cardiomyocytes increased TNFα mRNA expression and reduced IL-10 mRNA levels (Figure 7c). In contrast, the ANT1-TG-specific secretome did not induce a similar inflammatory cytokine expression pattern. Consequently, ANT1-TG cardiomyocytes were more stress-resistant than WT cells, and their secretome transferred these cell-protective properties to different cell types and influenced immunocompetent cells.

### 3.5. ANT1 Correlates with Immune System Regulatory Genes in the Hearts of Patients with Ischemic Cardiomyopathy 

Finally, the relation between ANT1 and genes related to the immune response was analyzed in left ventricular heart specimens from donors (*n* = 11) and patients with ischemic cardiomyopathy (*n* = 11). Microarray assay data published in the NCBI GEO database with the accession number GDS651 were used.

**Figure 8:**
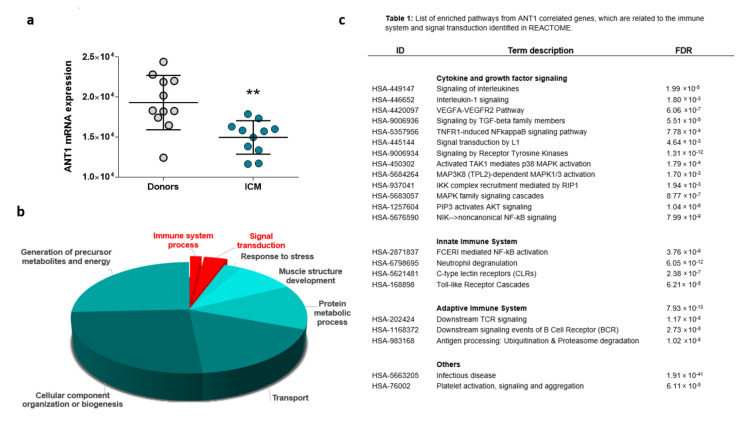
ANT1 correlating genes are involved in processes that regulate the immune response. Publicly available microarray data from the left ventricular heart tissue of donors and patients with ischemic cardiomyopathy (ICM) were used for Spearman’s correlation analysis to identify ANT1 (SLC25A4) co- and inverse-regulated genes. (**a**) Myocardial ANT1 transcription levels in controls and patients with ICM, ** *p* < 0.01. (**b**) Results of Gene Ontology (GO) enrichment analyses associated with ANT1-correlated genes; data are shown as −log (*p*)-values. (**c**) List of enriched pathways from ANT1 correlated genes, which are related to the immune system and signal transduction.

Compared with that in donor hearts, the *ANT1* mRNA level was reduced in heart tissue derived from ICM patients (Figure 8a), which corresponded to the reduced ANT1 expression in infarcted WT rat hearts (Figure 2a). Genes co-expressed with *ANT1* were determined by Spearman’s correlation analysis, which identified 646 positively correlated genes and 409 negatively correlated genes (Supplementary Table S1). The enrichment analysis of Gene Ontology (GO) terms using these genes showed that *ANT1*-correlating genes were primarily associated with oxidation-reduction processes, in addition to transportation, cellular organization, protein metabolism, muscle structure development, viral processes, and stress responses (Figure 8b).

A significant proportion of *ANT1*-linked genes were related to signal transduction and the immune system, influencing both adaptive and innate immunity (Figure 8c). *ANT1*-correlating genes affect cytokine and growth factor signaling, including IL-1β, TNF receptor 1 (TNFR1), VEGF, and TGFβ signaling. ANT1 correlating genes also influenced mitogen-activated protein kinase (MAPK) and protein kinase B (AKT) signaling pathways. 

## 4. Discussion

Previous studies have demonstrated that ANT1 overexpression has cardioprotective properties, affecting both intra- and extracellular signaling and increasing the release of immune-modulating factors. Therefore, we analyzed whether transgenic, heart-specific ANT1 overexpression influences the inflammatory process in infarcted heart tissue.

ANT1 overexpression promoted an anti-inflammatory immune response in infarcted hearts. The reduced pro-inflammatory immune response correlated with high mitochondrial integrity and a high survival rate, emphasizing the cardioprotective effects of the modulated immune response in ANT1-TG hearts. The shift in the immune response was promoted by the decreased transcription of pro-inflammatory cytokines and the increased expression and cellular release of anti-inflammatory immune modulators from ANT1-TG cardiomyocytes. The altered secretome composition supported the stress resistance of surrounding cells and prevented macrophages from expressing pro-inflammatory cytokines, such as TNFα. Finally, a close relationship was found between ANT1 and the immune response in human ischemic hearts, which underline the relationship between ANT1 expression and immunological processes in ischemic heart tissue.

### 4.1. ANT1 Overexpression Modulates the Immune Response in Ischemic Hearts

MI restricts ANT expression and function in both human and animal hearts [15,24]. The transgenic overexpression of ANT1 has been shown to compensate for this restriction, reducing the infarct size, and significantly improving the survival of infarcted ANT1-TG rats [15]. ANT1 overexpression has also been associated with the increased intactness of the mitochondrial inner membrane and the limited induction of oxidative stress compared with infarcted WT hearts. This study showed that ANT1-TG hearts displayed reduced pro-inflammatory IL-1β levels, correlated with high ANT1 and HSP27 expression, high mitochondrial integrity, and low oxidative stress levels. These findings inversely corresponded with our observations that IL-1β decreased ANT1 expression in isolated cardiomyocytes, known to damage mitochondrial function [25]. In addition, sham-operated ANT1-TG hearts expressed higher anti-inflammatory cytokine levels, such as IL-4, IL-10, and the growth factor, VEGF. The expression levels of IL-4 and IL-10 were elevated after MI in the ANT1-TG hearts but remained unchanged in infarcted WT hearts. IL-4 administration has been shown to enhance cardiac function, reduce infarct size, and increase tissue repair, supporting connective tissue and microvascular formation [26]. IL-10 treatment significantly decreased LV dilation, improved the ejection fraction, and reduced inflammation in murine MI hearts [27]. Furthermore, IL-10 significantly induces M2 macrophages and supports the regeneration of infarcted heart tissue. These findings support those from the present study, which showed that IL-4 and IL-10 expression levels correlated with cardiac M2 macrophage levels. Thus, ANT1-TG overexpression not only resulted in an altered cytokine expression pattern with a primarily anti-inflammatory character but also increased the presence of M2 macrophages. Sham-operated ANT1-TG hearts expressed increased levels of anti-inflammatory cytokines. However, the anti-inflammatory cytokine pattern did not result in a changed M1/M2 ratio of tissue macrophages in sham-operated hearts. Only after ischemia-induced tissue damage is the anti-inflammatory milieu in ANT1-TG hearts associated with an altered M1/M2 ratio, showing that immigrating or expanding macrophages react to the anti-inflammatory milieu in ANT1-TG hearts. Since we analyzed hearts early after infarction, we limited our studies to macrophages, which are the first immunocompetent cells infiltrating the damaged tissue, understanding that other immunocompetent cells may be part of the altered inflammatory process in ANT1-TG hearts. M2 macrophages additionally release anti-inflammatory cytokines, including IL-4, IL-10, TGFβ, and VEGF, which further support tissue regeneration. Simultaneously, the level of pro-inflammatory M1 macrophages was reduced in infarcted ANT1-TG hearts compared with that in WT hearts. Reduced myocardial M1 macrophage levels were positively correlated with reduced levels of intracellular stress, improved heart function, and an enhanced survival rate in ANT1-TG hearts, showing that the suppression of pro-inflammatory processes contributes to cardioprotection.

### 4.2. Interdependence of ANT1 and Cytokine Expression

Sham-operated ANT1-TG hearts showed higher levels of anti-inflammatory cytokines, including IL-10, IL-4, and VEGF-A. We, therefore, analyzed the interactions between ANT1 and cytokines in isolated cardiomyocytes, which proved that ANT1 transcription is sensitive to cytokine levels. Pro-inflammatory cytokines, such as TNFα/β, IL-6, IL-1β, and IFNs, significantly decreased the ANT1 mRNA levels in neonatal cardiomyocytes. TNFα is a pleiotropic cytokine that affects cell proliferation, metabolic activation, inflammatory response, and cell death. Pan et al. described a harmful effect for TNFα on ANT1 expression in myoblasts [28]. TNFα also induced RIP-dependent ANT inhibition, which reduced ATP production and necrotic cell death [29]. The transcription factor nuclear factor kappa B (NFκB) is a critical regulator of various pro-inflammatory cytokines. NFκB binds to two NFκB-responsive sites in the *ANT1* promoter and represses *ANT1* transcription, which impairs the ATP/ADP exchange [29]. Repressed ANT1 expression caused mitochondrial permeability transition pore opening and induces ROS production. Our previous studies corroborated a connection between ANT1 and NFκB. The phosphorylation of RelA/p65, one of the five NFκB constituents, was reduced and consequently less activated in ANT1-TG cardiomyocytes [16]. This reduced NFκB phosphorylation could explain the observed decrease in the expression of pro-inflammatory cytokines such as TNFα. The negative correlation between Rel and ANT1 transcription in the heart tissue of ICM patients additionally emphasizes the NFκB-dependent effect on ANT1 expression (Table S1). Anti-inflammatory IL-4 significantly increased ANT1 transcription in cardiomyocytes, similar to the effects observed for ANT3 in T helper cells [30]. Thus, pro-inflammatory cytokines decrease ANT1 expression and function in cardiomyocytes, which disrupt mitochondrial function. In contrast, anti-inflammatory cytokines support ANT1 expression and mitochondrial integrity.

Just as cytokines regulate ANT1 expression, ANT1 expression inversely influences cytokine and growth factor expression and release from cardiomyocytes. Transgenic ANT1 expression led to reduced IL-1α, TNFα, and TGFβ mRNA levels and increased VEGF expression. TGFβ promotes apoptosis and hypertrophy in cardiomyocytes, and our group has previously shown that TGFβ signaling is altered in adult ANT1-TG cardiomyocytes. ANT1-TG cardiomyocytes express less TGFβ receptor II than WT cells, whereas SMAD7 expression, an inhibitor of TGFβ signaling, was upregulated [31], resulting in the suppression of apoptosis-inducing processes in TGFβ-treated ANT1-TG cardiomyocytes. ANT1 knockdown has been linked to enhanced TNFα-induced NFκB reporter gene activity and IL-6 and TNFα expression in myocardium-derived H9c2 cells [28]. A mitochondrial-targeted antioxidant, mito-TEMPO, attenuated TNFα-induced mitochondrial ROS and cytokine expression in the ANT1 knockdown cells. Consequently, ANT1 reduction causes oxidative stress, which subsequently induces cytokine expression. In contrast, ANT1 overexpression reduces oxidative stress in oxygen-depleted cardiomyocytes and abates pro-inflammatory cytokine production [15].

ANT1-TG cardiomyocytes increase VEGF expression and secretion, which corresponds to an elevated level of the transcription factor hypoxia-inducible factor (HIF)1α, a VEGF regulator, in ANT1-TG cardiomyocytes [20]. VEGF plays a central role in angiogenesis, modulation of the macrophage response, and activation of the cell-protective mechanism. The pre-treatment of H_2_O_2_-treated H9c2 cardiomyocytes with VEGF reduced lactate dehydrogenase release, attenuated the decrease in the mitochondrial membrane potential and cytochrome c release, and consequently blocked H_2_O_2_-induced apoptosis [32]. Thus, high levels of extracellular VEGF have cell-protective effects on cardiomyocytes. Furthermore, the ANT1-TG cardiomyocyte-derived secretome could protect WT cardiomyocytes and endothelial cells against ischemic stress. This positive effect originates, at least in part, from the secretion of cell-protective VEGF and HSP27 from ANT1-TG cardiomyocytes [16]. The HSP27-mediated TLR4 pathway enhances ANT1 expression, stabilizes the mitochondrial membrane potential, and suppresses caspase 3 activity in cardiomyocytes. VEGF and HSP27 also support anti-inflammatory processes [17,33]. These data support our observation that the ANT1-TG-specific secretome inhibits the transcription of pro-inflammatory TNFα and the decrease in anti-inflammatory IL-10 in THP1 macrophages. In addition, Inia et al. discussed a model in which HSP27 communicates with macrophages via TLR4 signaling [34]. Thus, ANT1-TG cardiomyocytes suppress oxidative stress and NFκB activation and increase the release of immunomodulators and growth factors, which support stress resistance in different cell types and the modulation of cytokine expression among immunocompetent cells. Factors other than HSP27 and VEGF may contribute to the cell-protective and immunomodulating effects of the ANT1-specific secretome, which will be subject to further specific investigations.

### 4.3. ANT1 Transcription Correlates with Immune Response-Relevant Genes in the Hearts of Patients with Ischemic Cardiomyopathy

ANT1 mRNA levels were reduced in the explanted heart tissue of patients with ICM, which corresponded to the findings of restricted ANT1 expression and function observed in infarcted animal hearts [15,20]. In addition, ANT1 was found to correlate with genes that affect metabolic processes and are involved in the biogenesis, transport, contraction, gene expression, stress response, and viral infection processes. Previous studies demonstrated that transgenic ANT1 overexpression intervenes in the processes of energy metabolism, muscle structure and contraction [13,35], apoptosis, stress resistance [16,20,31], and viral infection [36]. Furthermore, ANT1 knockdown was associated with apoptosis, oxidative stress, and myocardial dysfunction in cellular and animal models [37,38]. Thus, the enrichment analyses performed on ANT1-correlating genes derived from ICM patients reflected several of these physiological processes in human heart tissue.

Mitochondrial dysfunction leads to the accumulation of metabolic components, including carbohydrates, lipids, and amino acids, affecting cytokine expression [3,39,40,41]. ANT1 correlated with genes involved in metabolic energy pathways. ANT1 overexpression compensates for metabolic dysfunction by stabilizing the mitochondrial membrane potential and the activity of oxidative phosphorylation (OxPHOS) complexes. The stabilized mitochondrial function suppresses oxidative stress [13,20], inhibiting the accumulation of metabolic components, and reducing the induction of inflammatory responses. A significant proportion of ANT1-correlating genes are involved in immunological processes, including the IL-1β, TNFα, TGFβ pathways, and the MAPK and TLR pathways. Other studies have indicated connections between ANT1 and these immune pathways. For example, TNFα-induced RIP-dependent ANT inhibition caused reduced ATP production and necrotic cell death [42]. Furthermore, ANT1-TG cardiomyocytes modulated TGFβ and MAPK pathways, reducing the apoptosis-inducing effects of TGFβ [20,31]. In addition, the activation of the HSP27-mediated TLR4 pathway increases ANT1 expression and supports the suppression of apoptosis [16]. Finally, the treatment of cardiomyocytes with IL-1β and TNFα reduced ANT1 transcription, as demonstrated in this study. Future molecular biological studies are required to clarify how cytokine-mediated transcription factors affect the ANT1 promoter, whose regulation is still largely unknown.

## 5. Conclusions

Mitochondrial function is linked to cytokine expression. ANT1 overexpression increases the expression of anti-inflammatory cytokines in control hearts. Mitochondrial function and ANT1 expression decrease in ischemic WT hearts, while ANT1 overexpression compensates for diminished ANT1 expression and stabilizes the mitochondrial function [15], which leads to reduced pro-inflammatory cytokine expression. Monocytes expand and/or migrate into an anti-inflammatory milieu and increasingly develop M2 characteristics after infarction. The increased myocardial level of M2-specific macrophages additionally supports the expression of anti-inflammatory cytokines and regenerative growth factors. These changes result in a cellular environment that attenuates ischemic tissue damage and provides the necessary conditions for rapid regeneration. The modulation of mitochondrial function by ANT1 affects cardiomyocytes and environmental cells, such as endothelial and immunocompetent cells, after MI. These findings demonstrate the significance of supporting mitochondrial function in infarcted hearts.

## Data Availability

Publicly available datasets were analyzed in this study. This data can be found here: https://www.ncbi.nlm.nih.gov, accessed on 10 August 2021, accession no. GDS651.

## Supplementary Materials

The following are available online at https://www.mdpi.com/article/10.3390/cells10082130/s1, Figure S1: Macrophage infiltration, Table S1: Genes that correlate with ANT1 (SLC25A4) in the heart tissue of heart donors and ICM patients.
