# Supplementary figures and images for: Adenine Nucleotide Translocase 1 Expression Modulates the Immune Response in Ischemic Hearts
Source: Cells. 2021 Aug 19;10(8):2130. doi: 10.3390/cells10082130 (PMC8393693; doi:10.3390/cells10082130)

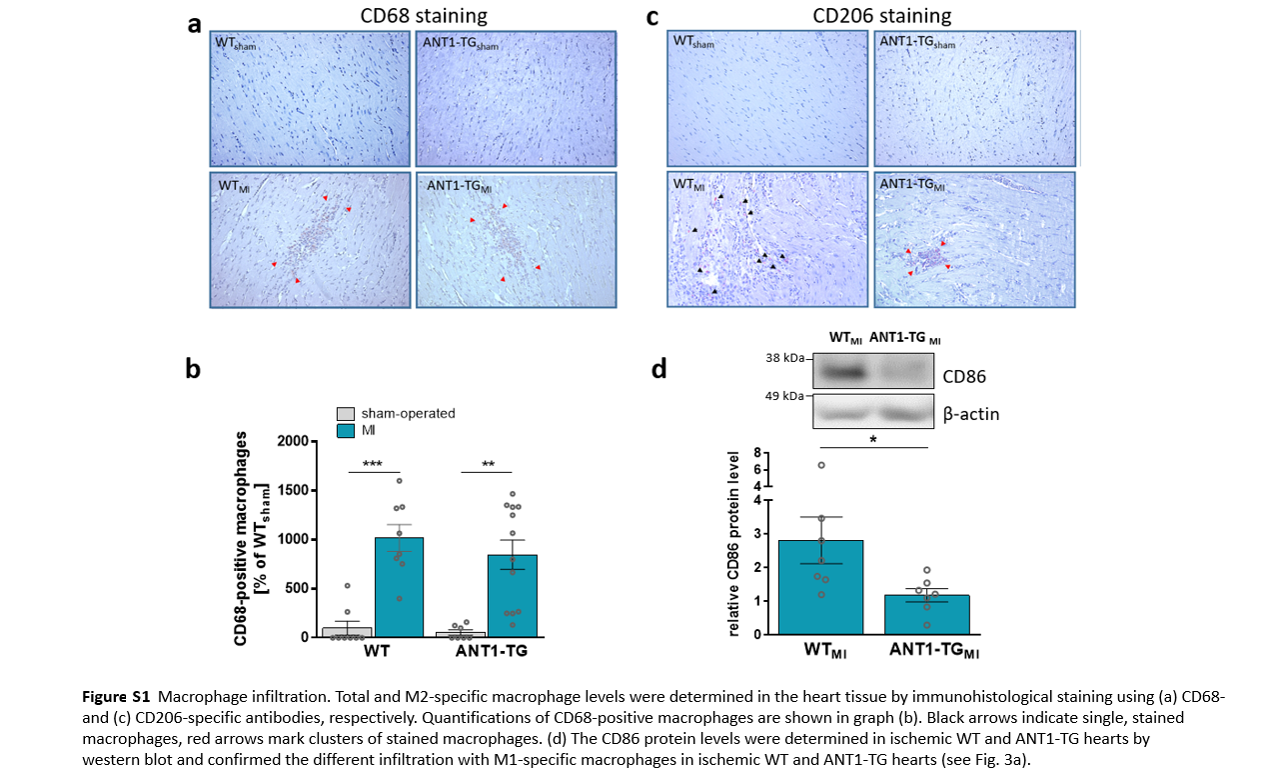

Supplement: Supplementary file 1 [file cells-10-02130-s001.zip › Figure S1.png]
